# DEC1 is involved in circadian rhythm disruption-exacerbated pulmonary fibrosis

**DOI:** 10.1186/s12964-024-01614-w

**Published:** 2024-04-26

**Authors:** Shuai-Jun Chen, Fan Yu, Xiao Feng, Qian Li, Ye-Han Jiang, Li-Qin Zhao, Pei-Pei Cheng, Meng Wang, Lin-Jie Song, Li-Mei Liang, Xin-Liang He, Liang Xiong, Fei Xiang, Xiaorong Wang, Hong Ye, Wan-Li Ma

**Affiliations:** 1 Department of Respiratory and Critical Care Medicine, Union Hospital, Tongji Medical College, Huazhong University of Science and Technology, 1277 JieFang Avenue, 430022 Wuhan, China; 2 Department of Pathophysiology, School of Basic Medicine, Tongji Medical College, Huazhong University of Science and Technology, 13 Hang Kong Road, 430030 Wuhan, China; 3 Key Laboratory of Respiratory Diseases, National Health Commission of China, Wuhan, China

**Keywords:** DEC1, p21, Pulmonary fibrosis, Alveolar epithelial type II cell

## Abstract

**Background:**

The alveolar epithelial type II cell (AT2) and its senescence play a pivotal role in alveolar damage and pulmonary fibrosis. Cell circadian rhythm is strongly associated with cell senescence. Differentiated embryonic chondrocyte expressed gene 1 (*DEC1*) is a very important circadian clock gene. However, the role of DEC1 in AT2 senescence and pulmonary fibrosis was still unclear.

**Results:**

In this study, a circadian disruption model of light intervention was used. It was found that circadian disruption exacerbated pulmonary fibrosis in mice. To understand the underlying mechanism, DEC1 levels were investigated. Results showed that DEC1 levels increased in lung tissues of IPF patients and in bleomycin-induced mouse fibrotic lungs. In vitro study revealed that bleomycin and TGF-β1 increased the expressions of DEC1, collagen-I, and fibronectin in AT2 cells. Inhibition of DEC1 mitigated bleomycin-induced fibrotic changes in vitro and in vivo. After that, cell senescence was observed in bleomycin-treated AT2 cells and mouse models, but these were prevented by DEC1 inhibition. At last, p21 was confirmed having circadian rhythm followed DEC1 in normal conditions. But bleomycin disrupted the circadian rhythm and increased DEC1 which promoted p21 expression, increased p21 mediated AT2 senescence and pulmonary fibrosis.

**Conclusions:**

Taken together, circadian clock protein DEC1 mediated pulmonary fibrosis via p21 and cell senescence in alveolar epithelial type II cells.

**Supplementary Information:**

The online version contains supplementary material available at 10.1186/s12964-024-01614-w.

## Background

Idiopathic pulmonary fibrosis (IPF) is a chronic, fibrosing interstitial pneumonia of unknown cause that is associated with radiological and histologic features of usual interstitial pneumonia (UIP). It is characterized by progressive lung function decline and lung structural distortion with a poor prognosis [1]. IPF occurs mainly in older individuals, with the average onset at about age 65 years and a mean survival time of 3–5 years after diagnosis.

Pathological fibrogenesis in IPF is a dynamic process involving complex interactions between epithelial cells, fibroblasts, immune cells (macrophages, T-cells), and endothelial cells. Chronic injury to alveolar epithelial type II cells (AT2) mediates disordered repair and abnormal activation of fibroblasts, driving disruption of lung architecture, massive extracellular matrix deposition, and eventual pulmonary fibrosis [2]. Thus, damage to the alveolar epithelium and abnormal wound repair are considered as key factors in IPF progression [3]. Reyfman PA utilized single-cell RNA sequencing and identified a cluster of AT2 cells that was from fibrotic lung and had an extraordinary high senescence score [4]. Yao and colleagues reported that AT2 cell senescence rather than loss of AT2 cells promoted progressive fibrosis [5]. Then, AT2 cell senescence plays a pivotal role in alveolar damage and fibrosis. However, the detailed mechanism of AT2 cell senescence in pulmonary fibrosis was still unclear.

Circadian rhythm is the 24-hour rhythm that organisms exhibit adapting to the earth′s rotation. Actions at all levels, from gene expression to behavior, are affected by the molecular circadian clock proteins. Moreover, the daily rhythm of cell is a cellular quality. In mammals, circadian rhythm of gene expression, cellular/organismal physiology and behavior is regulated by the circadian timing system. Any change in period or amplitude of the clock gene expression should result in circadian rhythm disorder [6–10]. The circadian clock exerts a profound influence on a myriad of physiological processes within the organism, including sleep patterns, dietary habits, metabolic rates, immune responses, and aging process [11–14]. Disruptions in circadian rhythm are recognized as significant indicators of aging [15–17]. Empirical evidence from extensive research utilizing mouse models has demonstrated that a disruption in the balance of core components of the circadian clock is associated with accelerated aging phenotypes [18–20]. Literature reports indicate an increase in the accumulation of senescent cells within various tissues, such as lung, liver, and spleen, in the clock gene knockout mice [21]. At the systemic level, dysfunction in circadian clock is identified as a critical risk factor for onset of various age-related diseases, encompassing neurodegenerative disorders, osteoarthritis, and metabolic syndromes [22–24]. In a word, these literatures underscore an inevitable link between the circadian clock function and cellular senescence. Differentiated embryonic chondrocyte expressed gene 1 (*DEC1*) is a very important component in the negative feed-back system of clock gene. *CLOCK, BMAL1, PER* and *CRY* are also clock genes. The basic mechanism of circadian rhythm is following: CLOCK and BMAL1 bind to E-boxes in the promoters of *DEC1*, *PER* and *CRY*, subsequently promote synthesis of their proteins. Increased-DEC1 combines with PER or CRY to form a dimmer which suppresses CLOCK and BMAL1 transactivation. So, dimmer CLOCK/BMAL1 and DEC1/PER (or DEC1/CRY) form negative feedback which present as circadian rhythm [25–27].

As described above, AT2 cell senescence plays a pivotal role in alveolar damage and fibrosis. Cell circadian rhythm is strongly associated with cell senescence. Consequently, the influence of circadian rhythm on cell senescence and its further impact on pulmonary fibrosis warrants thorough investigation. This study revealed that disruption of the circadian rhythm intensified bleomycin-induced pulmonary fibrosis in mice. We aimed to determine the role of DEC1 in modulating senescence in alveolar epithelial cells and its potential contribution to the progression of pulmonary fibrosis.

## Results

### Circadian rhythm disruption exacerbated pulmonary fibrosis in mice

**Fig. 1:**
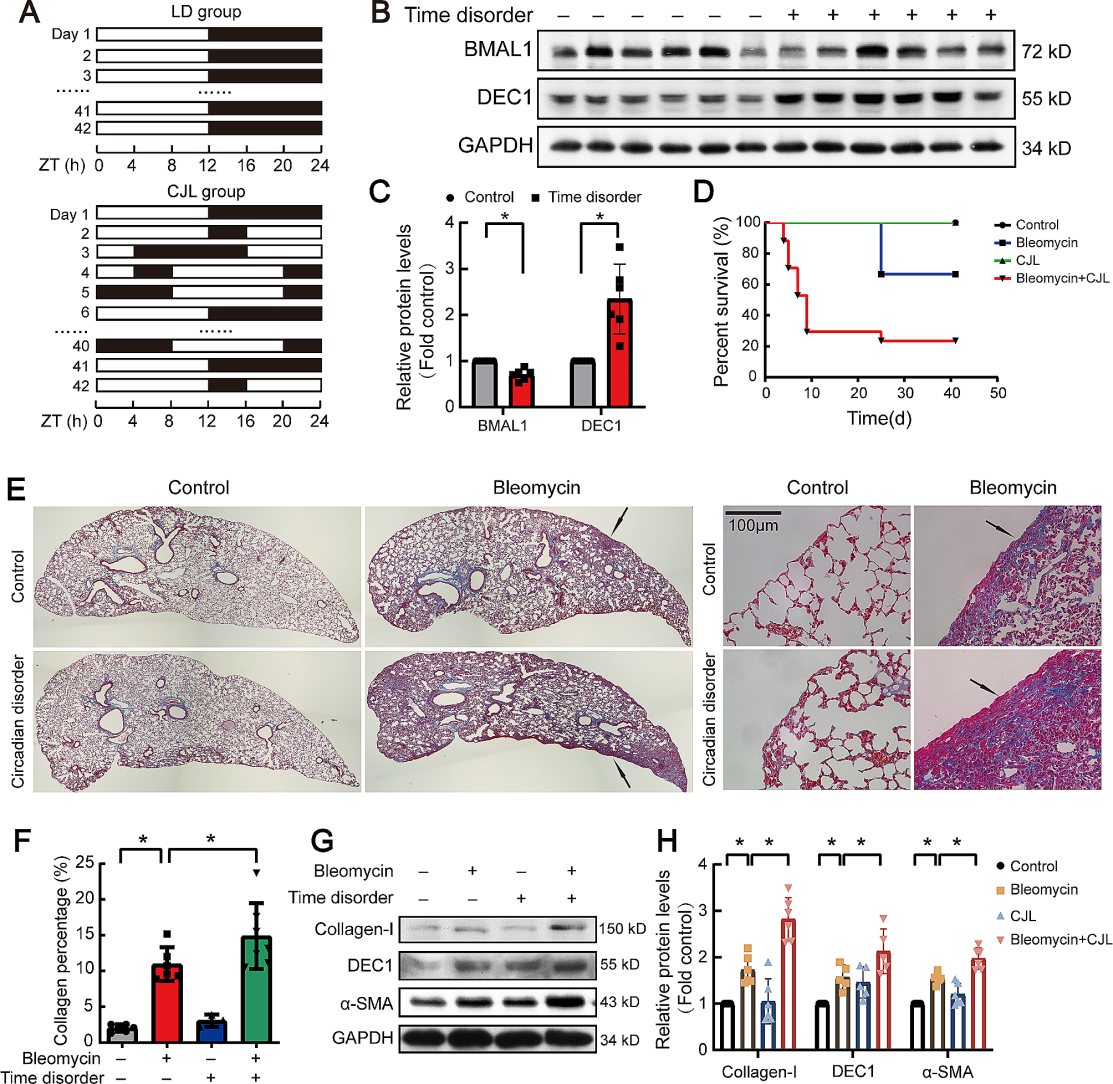
Circadian rhythm disruption exacerbated pulmonary fibrosis in C57BL/6J mice. All C57BL/6J mice were synchronized to a light dark cycle with 12 h of light and 12 h of darkness (LD) for 2 weeks. Then the CJL group was treated by advancing light onset 8 h in every 48 h, 5 days in rhythm disruption was exactly a cycle. The LD group was maintained at normal circadian rhythms. (**A**) Schematic diagram of animal model for disrupted circadian rhythms. (**B, C**) BMAL1 and DEC1 proteins were measured by western blotting, changes in relative density of BMAL1 and DEC1 were presented. *n* = 6. (**D**) Survival analysis of mice in each group. (**E**) Representative images of lung tissue with Masson staining. Original magnification, ×400. (**F**) Collagen deposition area (%) of lung tissue in each group. *n* = 6. (**G**) Representative immunoblots of collagen-I, DEC1 and α-SMA proteins in lung lysates. (**H**) Changes in protein levels of collagen-I, DEC1 and α-SMA (*n* = 5). Data are shown as mean ± SEM of n individual experiments. *P < 0.05, *P* values were determined by Student’s *t*-test (**C**) or one-way ANOVA followed by the Bonferroni’s test (**F, H**)

In order to elucidate the role of circadian rhythm in pulmonary fibrosis, we established a jet-lagged mouse model to make circadian rhythm disruption. C57BL/6J mice were placed at a jet lag schedule with 8 h-light advanced every 48 h, mimicking circadian disturbance that human being undergoes during shift work. The detailed jet lag schedule of animal model is shown in Fig. 1A. 41 days later, expression of DEC1 was detected. As shown in Fig. 1B and C, circadian rhythm disruption increased DEC1 protein in mouse lung tissue, while expression of BMAL1 protein decreased. Interestingly, compared with mice in the bleomycin group, the survival percent in the group of bleomycin plus circadian rhythm disruption was significantly reduced (Fig. 1D). Morover, conventional Masson staining and western blot showed that the circadian rhythm disruption aggravated deposition of extracellular matrix (ECM) in mouse lung (Fig. 1E-H), suggesting that circadian rhythm disruption was involved in pulmonary fibrosis.

### DEC1 expression increased in lung tissue of IPF patients and bleomycin-induced mouse fibrotic lungs

**Fig. 2:**
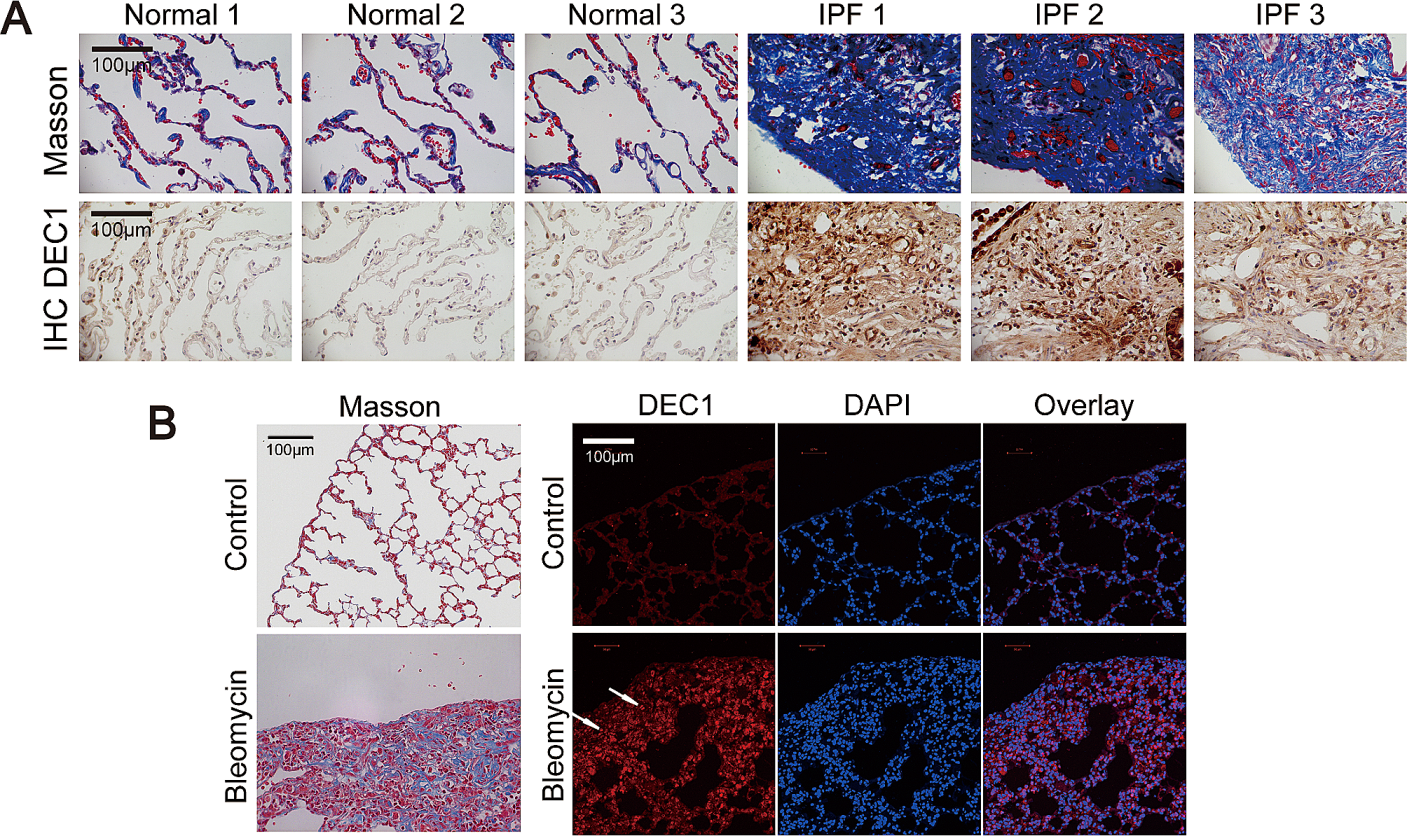
DEC1 expression increased in IPF patient′s lung tissues and bleomycin induced C57BL/6J mouse fibrotic lungs. (**A**) Representative images of Masson staining and DEC1 protein immunohistochemistry of lung tissue from IPF patients. The control was adjacent normal lung tissues of lung cancer. Original magnification, ×400. (**B**) C57BL/6J mouse pulmonary fibrosis model, representative images of DEC1 protein immunofluorescence staining and Masson staining of lung tissues. Original magnification, ×400

By immunohistochemistry, DEC1 expression in lung tissue of IPF patients was detected. Compared with control lung tissues, DEC1 was highly expressed in lung tissues of IPF patients (Fig. 2A). At the same time, expression of DEC1 was also detected in mouse pulmonary fibrosis models, and same results were obtained (Fig. 2B).

**Fig. 3:**
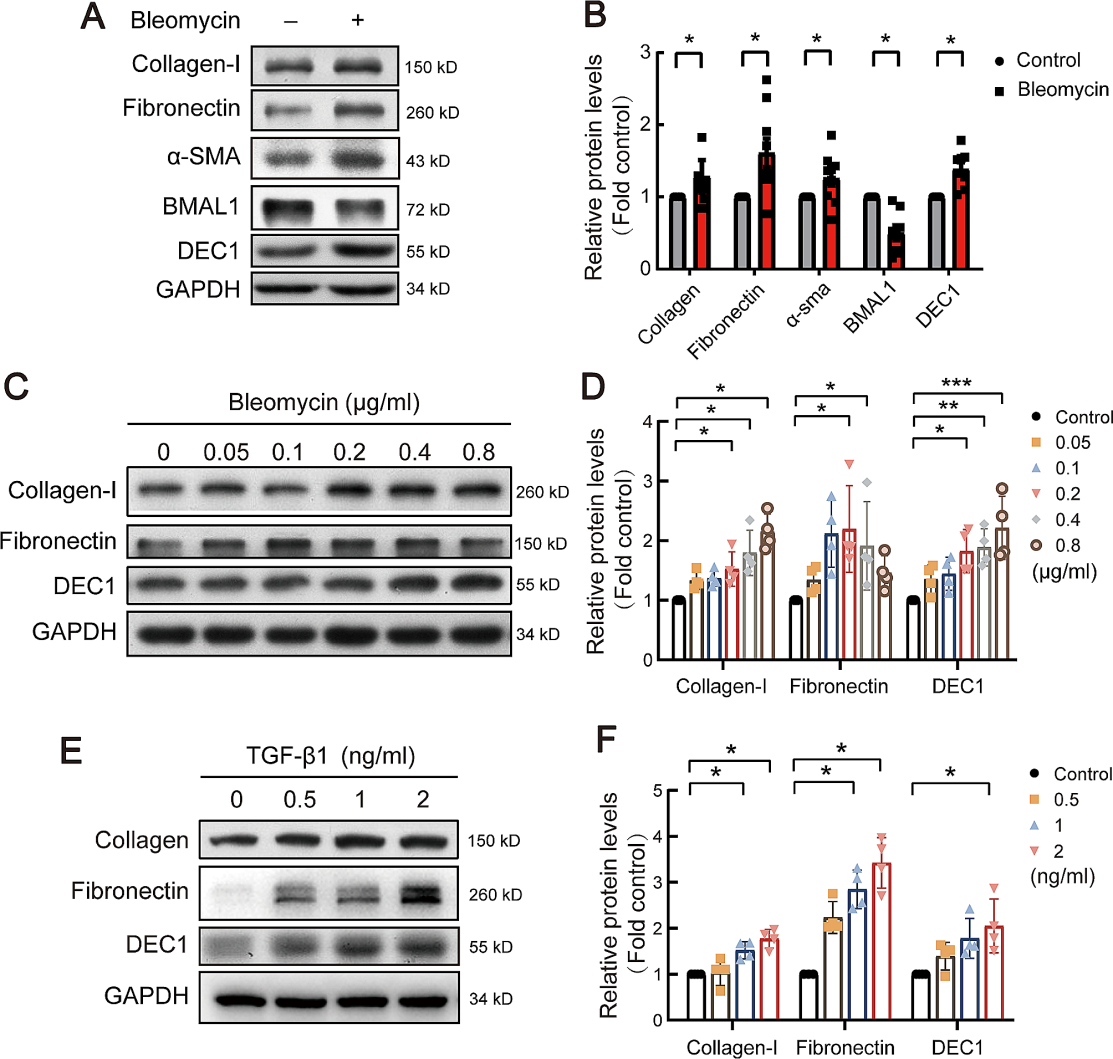
Bleomycin and TGF-β1 increased expressions of collagen-I, fibronectin and DEC1 in rat alveolar epithelial cells. (**A, B**) Primary alveolar epithelial cells were treated with bleomycin (0.2 µg/ml) for 48 h, and Western blot was performed to detect collagen-I (*n* = 6), fibronectin (*n* = 7), α-SMA (*n* = 10), BMAL1 (*n* = 8) and DEC1 (*n* = 8) proteins. (**C-F**) Serial concentrations of bleomycin or TGF-β1 (2 ng/ml) were used to treat RLE-6TN cells. The expression of related proteins, collagen-I, fibronectin and DEC1, was detected by Western blot. The representative images of immunoblots and statistical analysis were shown. *n* = 4. *P < 0.05, **P < 0.01, ***P < 0.001. *P* values were determined by Student’s *t*-test (**B**) or one-way ANOVA followed by the Dunnett’s test (**D, F**)

Primary mouse type 2 alveolar epithelial cells were used to further verify the expression of DEC1 in vitro. The extracted primary AT2 cell was verified by electron microscopy (Fig. S1). As shown in Fig. 3A and B, after treating primary cells with bleomycin, the expression of DEC1 increased accompanied with changes of fibrosis-related proteins collagen-I, fibronectin and α-smooth muscle actin (α-SMA). Moreover, type 2 alveolar epithelial cell line RLE-6TN cells were treated with serial concentration of bleomycin, it revealed that DEC1 protein presented dose-dependent changes (Fig. 3C-D). TGF-β1 is the key factor in lung fibrosis, as similar as bleomycin, TGF-β1 also induced dose-dependent increases of DEC1 protein.

These data indicated that DEC1 expression up-regulated in the lung of pulmonary fibrosis in vivo and in vitro.

### DEC1 inhibition attenuated bleomycin-induced pulmonary fibrosis in mouse model

**Fig. 4:**
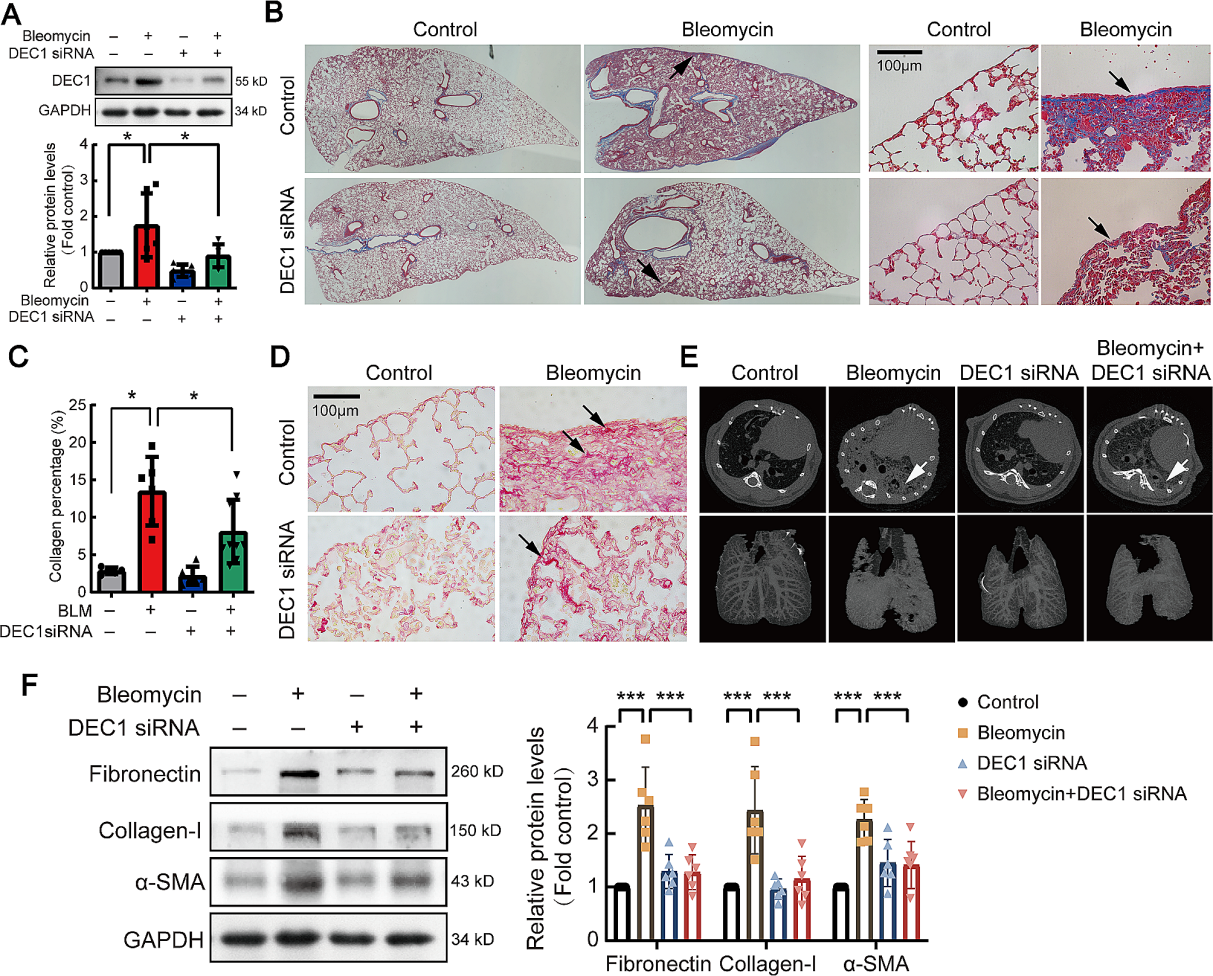
DEC1-siRNA suppressed bleomycin-induced mouse pulmonary fibrosis. C57BL/6J mice were subjected to intraperitoneal injection of DEC1 siRNA lentivirus to knock down DEC1 gene, and then a pulmonary fibrosis model was established by intraperitoneal injection of bleomycin. (**A**) Efficiency validation of DEC1 interference in mouse lung tissue (*n* = 5). (**B**) A panoramic and 400-times magnified representative images of Masson staining of mouse lung tissue. (**C**) Quantitative analysis of collagen deposition area in mouse lung tissue (*n* = 6). (**D**) Sirius red staining of mouse lung tissues. (**E**) Micro-CT scan image and 3D reconstruction of mouse lung. (**F**) Western blot analysis of fibronectin, collagen-I, and α-SMA in mouse lung tissue (*n* = 6), quantitative analysis of fibronectin, collagen-I, and α-SMA protein blots. *P < 0.05, **P < 0.01, ***P < 0.001. *P* values were determined by the one-way ANOVA followed by the Bonferroni’s test

To explore the role of up-regulated DEC1 in pathogenesis of pulmonary fibrosis, DEC1 siRNA lentivirus was constructed to knock down DEC1 expression in mouse lung tissues. As shown in Fig. 4A, the virus induced depression of DEC1 in RLE-6TN cells, and restrained bleomycin induced up-regualtion of DEC1. In mouse pulmonary fibrosis models, Masson and Sirius red staining showed that collagen deposition in lung tissue was inhibited by DEC1 siRNA (Fig. 4B-D). Prevention effect of DEC1 siRNA in mouse pulmonary fibrosis also exhibited by small animal lung CT scans and 3D imaging of lungs (Fig. 4E). At the same time, fibrosis-related proteins fibronectin, collagen-I and α-SMA in lung tissue were investigated, bleomycin increased these proteins, but the increased-proteins were restrained by DEC1 siRNA (Fig. 4F). Therefore, our data suggested that inhibition of DEC1 attenuated bleomycin-induced lung fibrosis.

**Fig. 5:**
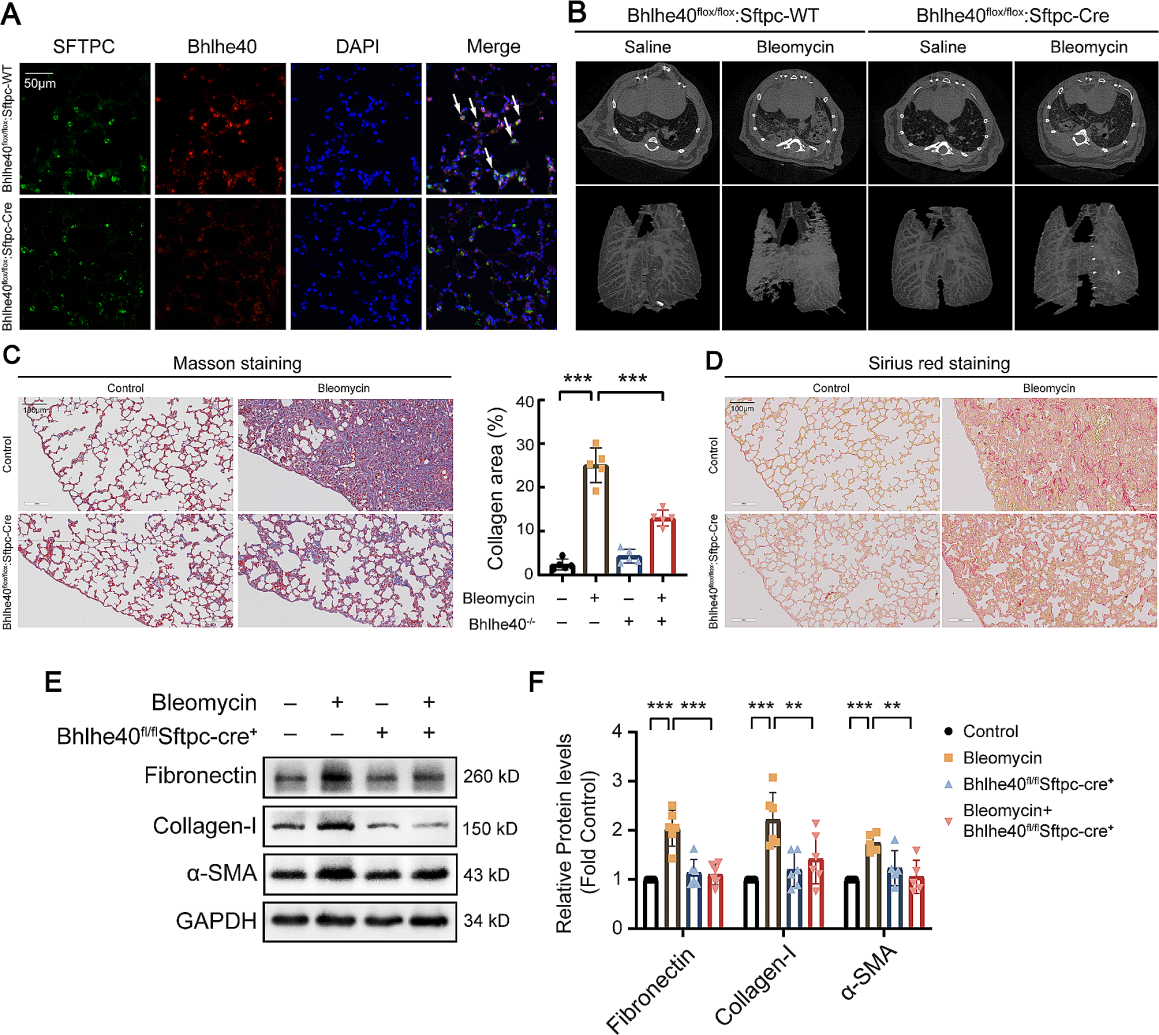
Mouse type II alveolar epithelial cell specific knockout of DEC1 attenuated bleomycin-induced mouse pulmonary fibrosis. (**A**) Representative images of DEC1 gene knockout in mouse alveolar epithelial cells were verified by immunofluorescence. Original magnification, ×400. (**B**) DEC1 conditional knockout mice were used to construct a pulmonary fibrosis model. The figure showed CT scan and 3D reconstruction images of mouse lung. (**C**) Masson staining of lung tissues. Original magnification, ×200. (**D**) Sirius red staining of lung tissues. Original magnification, ×200. (**E, F**) Representative blots showing the expression level of related proteins in lung tissue of mice, detected by Western blot analysis (*n* = 6). *P < 0.05, **P < 0.01, ***P < 0.001. *P* values were determined by the one-way ANOVA followed by the Bonferroni’s test

Next, type 2 alveolar epithelial cell specific DEC1 conditional knockout mice were constructed. As shown in Fig. 5A, DEC1 was almost knocked out in mouse type 2 alveolar epithelial cells. The pulmonary fibrosis model was made in DEC1 conditional knockout mice. As shown in Fig. 5B, after knockout of DEC1, CT scan and imaging showed that fibrotic area of lung tissue greatly reduced. At the same time, Masson and Sirius red staining were performed on the lung tissue of mice, and it was found that collagen deposition was significantly reduced compared with the bleomycin-control group (Fig. 5C and D). Western blot analysis of mouse lung tissue revealed similar results, which showed DEC1 knockout restrained bleomycin-induced up-regulation of fibronectin, collagen-I and α-SMA in mouse lung (Fig. 5E and F).

### Cell senescence increased in bleomycin-treated alveolar epithelial cells and pulmonary fibrosis models

**Fig. 6:**
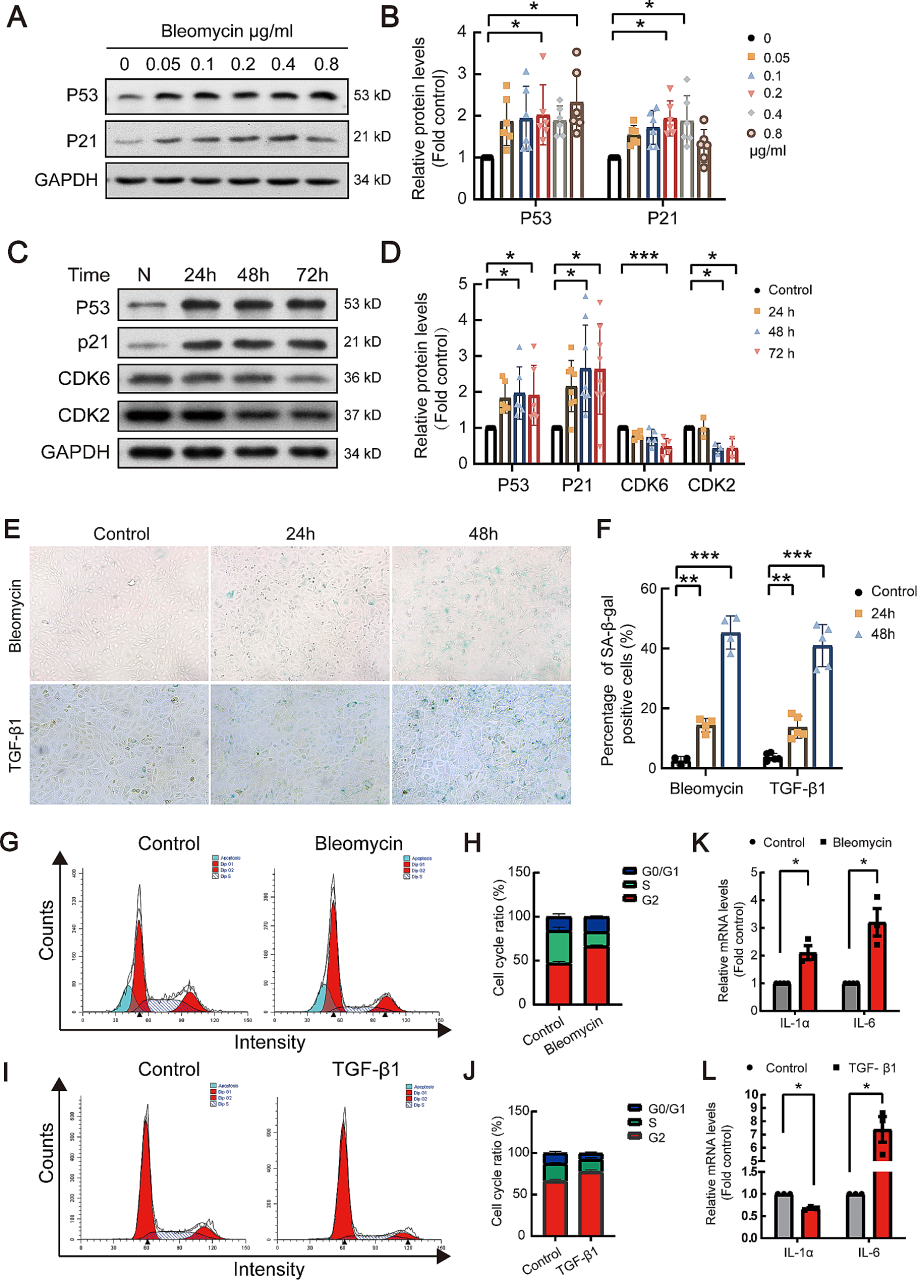
Bleomycin and TGF-β1 induced cell senescence in type II alveolar epithelial cells. (**A, B**) RLE-6TN cells were treated with a serial concentration of bleomycin, and Western blot analysis was performed to detect protein levels of p21 and p53. (**A**) representative blots. (**B**) Statistical analysis, *n* = 6. (**C, D**) After treatment with bleomycin for a serial of time points, Western blot was used to detect the protein expression of p53, p21, CDK6, and CDK2 in RLE-6TN. Statistical analysis was conducted. (**E, F**) Cells were treated with bleomycin (0.2 µg/ml) or TGF-β1 (2 ng/ml), and cell senescence levels were assessed by β-galactosidase assay. (**G-J**) Bleomycin or TGF-β1 was used to treat cells for 24 h, changes in cell cycle were detected by flow cytometry. (**K, L**) Senescence-related gene miRNA levels were tested using qRT-PCR. *P < 0.05, **P < 0.01, ***P < 0.001. *P* values were determined by the one-way ANOVA followed by the Bonferroni’s test

Cell senescence was considered relating to pulmonary fibrosis. Studies have shown that senescent fibroblasts and alveolar epithelial cells are involved in the pathogenesis of pulmonary fibrosis. Therefore, expression levels of aging markers p21 and p53 were detected in alveolar epithelial cells in vitro. As shown in Fig. 6A-D, after alveolar epithelial cells were treated with bleomycin, protein levels of p53 and p21 increased, on the contrary, protein levels of cyclin CDK6 and CDK2 decreased. Using the β-galactosidase assay, it was found bleomycin or TGF-β1 increased the proportion of senescence cells (Fig. 6E and F). Furthermore, treatment of bleomycin or TGF-β1 blocked cells in the G1-S phase of cell cycle (Fig. 6G-J). Bleomycin or TGF-β1 increased IL-1α and IL-6 levels which indicated the cells were senescence-associated secretory phenotypes (SASP) (Fig. 6K and L). Above results suggested that bleomycin and TGF-β1 promoted senescence of alveolar epithelial cells in vitro.

Next, expression levels of p21 and p53 were detected in mouse pulmonary fibrosis models. As shown in online Fig. S3, bleomycin increased expressions of p21 and p53 protein. The results indicated that bleomycin promoted cell senescence in pulmonary fibrosis in vivo.

### DEC1 mediated cell senescence in pulmonary fibrosis

**Fig. 7:**
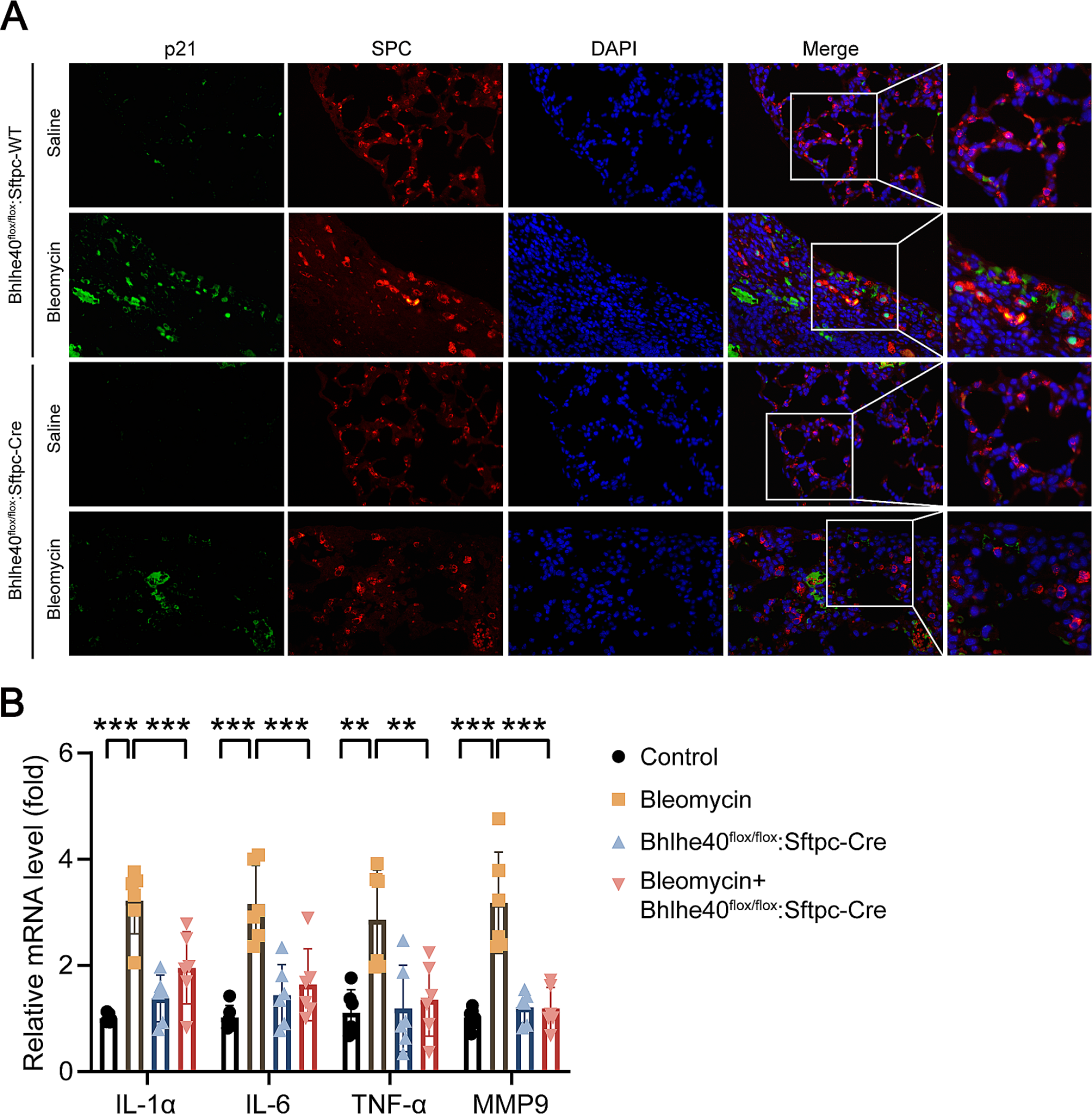
Mouse alveolar epithelial cell specific knockout of DEC1 attenuated ATII cell senescence in bleomycin-induced mouse pulmonary fibrosis. (**A**) Representative immunofluorescence images of p21 protein expression in pulmonary fibrosis models constructed in mice with or without DEC1 knockout. Original magnification, ×400. Green: p21, red: SFTPC. (**B**) qRT-PCR was performed to detect mRNA levels of the senescence-associated secretory phenotype (SASP) factors, including IL-1α, IL-6, TNF-α, and MMP-9 in mouse lung tissues (*n* = 6). *P < 0.05, **P < 0.01, ***P < 0.001. *P* values were determined by the one-way ANOVA followed by the Bonferroni’s test

To verify relationship between DEC1 and alveolar epithelial cell senescence, DEC1 conditional knockout mouse models and cell senescence were investigated. As shown in Fig. 7A, the protein level of p21 in alveolar epithelial cells of DEC1 conditional knockout mice was significantly lower than that of the bleomycin group. RT-qPCR showed that DEC1 knockout inhibited bleomycin-induced increases of mRNAs level of SASP related proteins in lung tissue (Fig. 7B). Usage of DEC1 siRNA in mice also prevented bleomycin-induced alveolar cell senescence in pulmonary fibrosis models (Fig. S2). These data suggested that DEC1 induced alveolar cell senescence in pulmonary fibrosis model.

**Fig. 8:**
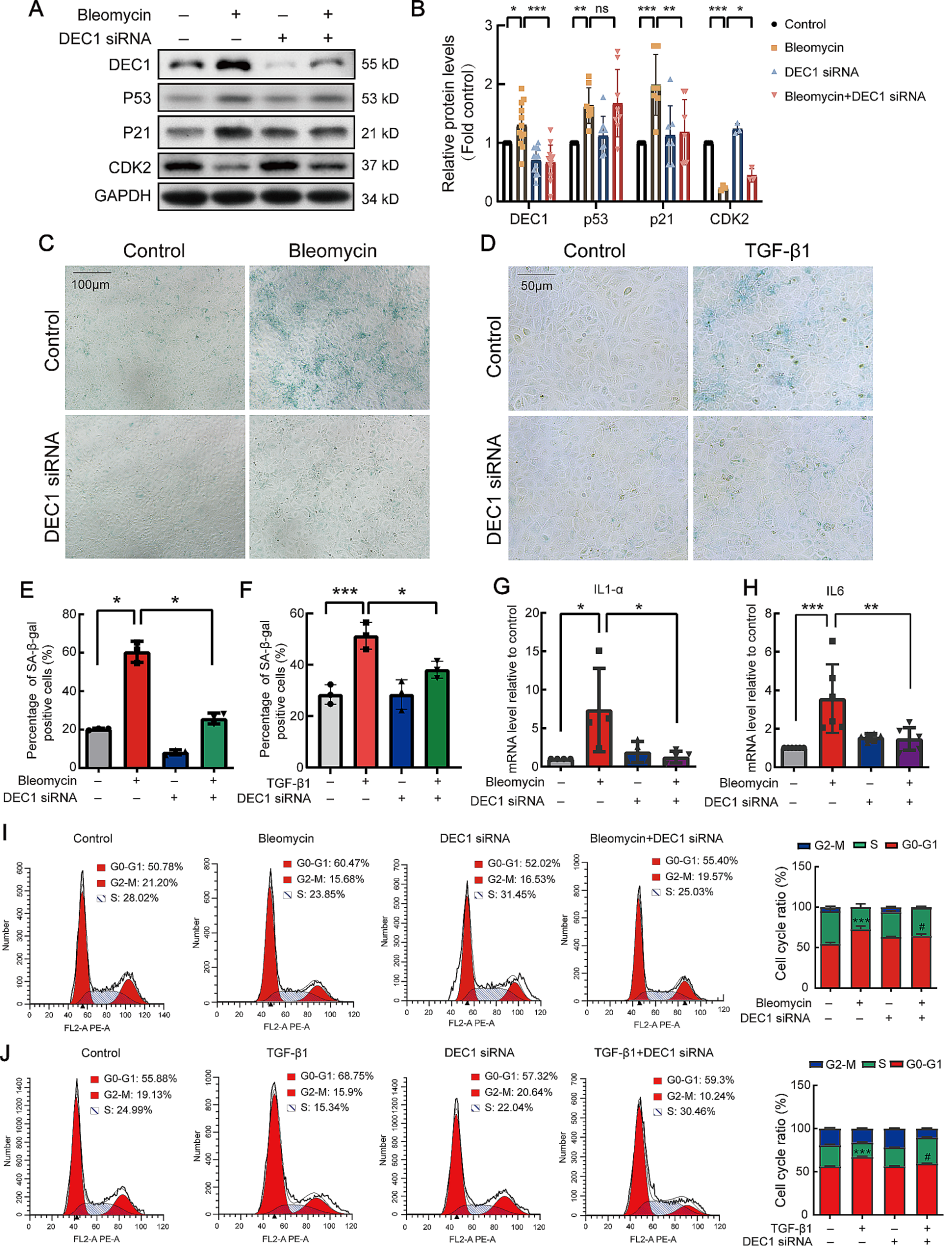
Knockdown of DEC1 with DEC1 siRNA attenuated bleomycin or TGF-β1 induced ATII cell senescencein vitro. (**A**) RLE-6TN cells were treated with bleomycin and DEC1 siRNA, and the expression levels of senescence-associated proteins DEC1, p53, p21, and CDK2 were detected by Western blot analysis. (**B**) Statistical analysis of the Western blot bands corresponding to the senescence-associated proteins detected. DEC1: *n* = 12, p53: *n* = 8, p21: *n* = 6, and CDK2: *n* = 3. (**C, D**) After treating RLE-6TN cells with bleomycin or TGF-β1 with or without DEC1 siRNA, cellular senescence was detected by staining with β-galactosidase. (**E, F**) Statistical analysis of senescent cells in according to C and D, *n* = 3. (**G, H**) SASP (IL-1α and IL-6) in epithelial cells was detected by qRT-PCR after treating RLE-6TN cells with bleomycin with or without DEC1 siRNA (G: *n* = 4, H: *n* = 6). (**I, J**) RLE-6TN cells were treated with bleomycin or TGF-β1 with or without DEC1 siRNA, and changes in the cell cycle were detected by flow cytometry, *n* = 3. *P < 0.05, **P < 0.01, ***P < 0.001, ns means nonsignificant. *P* values were determined by the one-way ANOVA followed by the Bonferroni’s test

As shown in Fig. 8A and B, DEC1 siRNA administration notably attenuated the escalation of the senescence marker p21 induced by bleomycin, yet exhibited negligible impact on p53 expression. Conversely, knockdown of p21 elucidated no discernible mediation of DEC1 protein expression (Fig. S4). β-galactosidase assay exactly revealed that bleomycin- and TGF-β- induced cell senescence was alleviated by DEC1 siRNA (Fig. 8C-F). Moreover, as the SASP cytokines, IL1-α and IL-6 production reduced after DEC1 siRNA transfection in alveolar epithelial cells (Fig. 8G and H). DEC1 siRNA also restored cell cycle arrest induced by bleomycin (Fig. 8I and J). These results indicated that DEC1 mediated alveolar epithelial cell senescence in vivo.

### DEC1 mediated cell senescence via p21 in pulmonary fibrosis

**Fig. 9:**
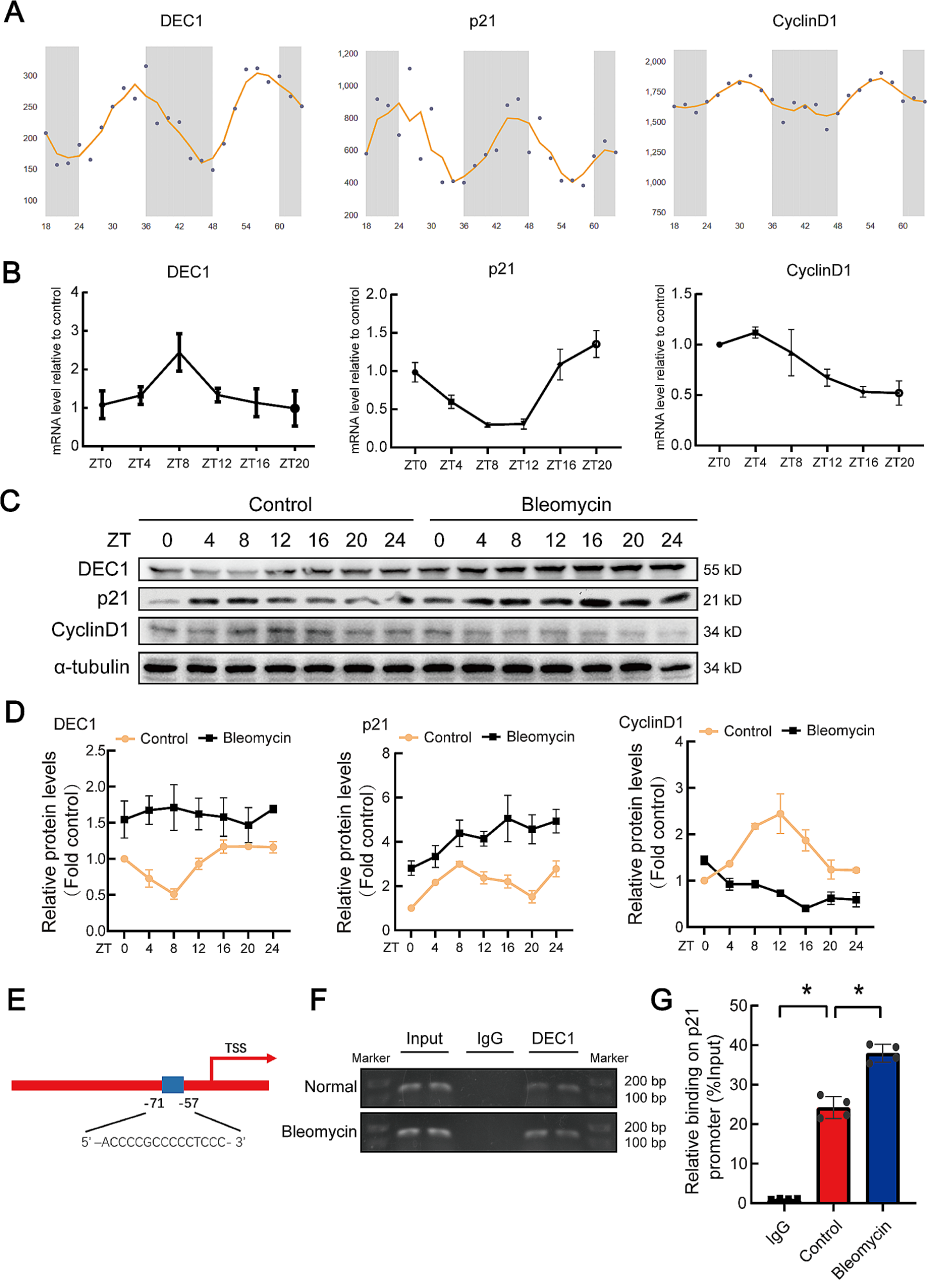
The cell cycle is rhythmical, and bleomycin disrupts the cell cycle rhythm in alveolar epithelial cells. (**A**) The website predicted the rhythms of circadian clock proteins and related cell cycle protein (Circadian Expression Profiles Data Base: http://circadb.hogeneschlab.org/). (**B**) The rhythms of the mRNA levels of circadian clock genes and cell cycle genes were detected in C57 mouse lung tissue by qRT-PCR. *n* = 6. (**C, D**) After treat RLE-6TN cells with bleomycin, Western blot analysis was performed to detect the rhythmic change of related proteins, and statistical analysis of blots was conducted. *n* = 3. (**E**) Schematic illustration of the binding sites between DEC1 and p21 promoter regions. (**F**) DNA electrophoresis gels showed PCR products obtained after the reaction with ChIP-enriched DNA. (**G**) ChIP-qPCR was used to quantitatively detect the binding of DEC1 to the p21 promoter region. *n* = 4. *P < 0.05. *P* values were determined by the one-way ANOVA followed by the Bonferroni’s test

Next, mechanism of DEC1 mediating cell senescence was investigated. Through analysis of online profiles of circadian rhythm expressions, DEC1, p21 and cyclingD1 presented periodic rhythm (Fig. 9A). In current study, the rhythmic expression of p21 and cyclinD1 in lung tissue was detected by RT-qPCR in vivo (Fig. 9B). We also did protein time-changes investigation. As shown in Fig. 9C and D, in control situation, DEC1 and p21 had circadian rhythm in vitro. After treatment with bleomycin, levels of DEC1 and p21 expression only increased instead of circadian rhythm. Through the Jasper website, it predicted that DEC1 had a binding site in the p21 promoter region (Fig. 9E). CHIP-qPCR experiments verified that DEC1 did bind to the p21 promoter region, and after bleomycin treatment, DEC1 had an increased ability to bind and promote p21 transcription (Fig. 9F and G). Therefore, DEC1 mediated cell senescence by transcriptionally regulating p21.

## Discussion

In this study, chronic jet lag (CJL), a circadian disruption model of light intervention was used [28]. It was firstly found that CJL exacerbated pulmonary fibrosis in mice. To understand the underlying mechanism, DEC1 levels were detected. Results showed that DEC1 levels increased in lung tissues of IPF patients and bleomycin-induced mouse fibrotic lungs. In vitro study revealed that bleomycin and TGF-β1 increased expressions of DEC1, collagen-I and fibronectin in cultured primary rat alveolar epithelial cells. Inhibition of DEC1 mitigated bleomycin-induced fibrotic changes in vitro and in vivo. After that, cell senescence was found in bleomycin-treated alveolar epithelial cells and mouse models, but these were prevented by DEC1 inhibition. Through analysis on the circadian rhythm website, it is postulated that the cell senescence marker p21 exhibits a circadian oscillation, which is perturbed following in vitro exposure to bleomycin. Our investigation culminated in the elucidation of the circadian clock’s role in mediating the transcriptional control of p21 via DEC1, consequently driving alveolar epithelial cell senescence and pulmonary fibrosis.

In a state of unperturbed physiological equilibrium, the lung exhibits a robust circadian rhythm, characterized by peak functionality occurring at midday (12:00) and nadir performance in the early hours of the morning (04:00) [29, 30]. This rhythmicity extends to various cellular components within lung tissue, notably the alveolar epithelium [31]. However, this circadian rhythm perturbations which induced by a spectrum of environmental stimuli such as light exposure, lipopolysaccharide (LPS) challenge, tobacco smoke inhalation, and administration of bleomycin precipitated the progression of chronic pulmonary pathologies [32, 33]. Given the lung’s pivotal role as an interface with the external milieu, circadian regulation disruptions assume heightened significance in the etiology and exacerbation of respiratory disorders. Cunningham PS and colleagues reported that fibrotic mouse lungs exhibited disorder of circadian rhythm in bleomycin-induced lung fibrosis [34]. Our study revealed that circadian rhythm disturbances exacerbated pulmonary fibrosis in mice. Furthermore, we observed elevated DEC1 expression in alveolar epithelial cells within lung tissues of both idiopathic pulmonary fibrosis (IPF) patients and a bleomycin-induced pulmonary fibrosis mouse model.

Pulmonary fibrosis, especially IPF is an age-related disorder. During the process of pulmonary fibrosis, damaged ATII cells acquired a senescent phenotype, producing cell cycle arrest, SASP factors, leukotrienes, and prostaglandins. These factors collectively contributed to proliferation of myofibroblasts which were responsible for aberrant deposition of ECM [35]. These senescent cells exerted autocrine and paracrine effects which led to neighboring cells senescence. More and more SASP damaged surfactant functions of ATII cells, and induced collapse of the alveolus. So, stress-induced premature senescence of ATII cells and chronic SASP are key regulators of pulmonary fibrosis [36, 37]. Cell senescence was characterized by cell-cycle arrest in the G1 or possibly G2 phase, which prevented proliferation of damaged cells. DNA damage activated p53/p21 signaling pathway, and p21 was mainly activated during early stage of cell senescence [38]. In our study, we found that bleomycin enhanced senescence in alveolar epithelial cells, inducing G1 phase arrest. DEC1 knockdown mitigated this bleomycin-induced senescence. Notably, cyclins (p21, cyclinD1) exhibited a periodic rhythm, disrupted by bleomycin, leading to sustained high levels of p21. This rhythmic disruption was confirmed via CHIP-PCR, showing DEC1’s transcriptional regulation of p21. Literature in recent years aligns with our observations, highlighting that cell cycle processes exhibit circadian rhythms, modulated by core circadian clock proteins [39–42].

The cell cycle is predominantly governed by the Rb and p53/p21 signaling pathways, which are interconnected. p53, acting as a transcription factor, can regulate p21 expression by binding to its promoter region, thereby influencing cell cycle progression. Our findings suggested that DEC1 primarily mediated the transcriptional regulation of p21, inducing cell cycle arrest, while not affecting p53. This implied that in pulmonary fibrosis, DEC1 facilitated alveolar epithelial cell senescence through p21 regulation, independent of p53. Given that both DEC1 and p53 can target the p21 promoter to modulate the cell cycle, the potential for competitive inhibition warrants further investigation.

In addition to the above discussion, DEC1 contributed to pulmonary fibrosis also through immunity regulation. DEC1 was a key regulator of alveolar macrophage self-renewal and guardians of their identity [43], and promoted macrophage pro-inflammatory gene expression and functions [44]. DEC1 fostered tissue resident memory T cell commitment and activity which was involved fibrosis [45, 46]. Whether DEC1 regulates immunity through p21 needs to be studied in the future.

In conclusion, our data provided evidences that DEC1 up-regulated in type II alveolar epithelial cells of fibrotic lung. DEC1 regulated cell cycle rhythm and increased p21 which led to ATII cell senecence and SASP release, as well as contributed to occurrence of pulmonary fibrosis.

## Methods

### Isolation and culture of rat type II alveolar epithelial cells

Primary human AT2 cells were isolated directly from peripheral lung tissue employing a specialized technique [47]. After anesthesia, the skin of the rat was disinfected with 75% ethanol and cut from the abdomen to the mouth with scissors on the operating table to expose the neck and thorax. Then the peritoneum was cut lengthwise and the abdominal aorta was cut and the blood was released. Cut the thorax through the diaphragm and remove the sternum, fully exposing the lung tissue. The thymus was removed with tweezers, then the base of the heart was gently picked up and 10 ml of PBS was injected into the right atrium, thereby whitening the lung tissue and reducing the effect of the number of red blood cells on the final type II alveolar epithelial cell production and purity. Lung tissue was excised and immediately immersed in precooled CNT-17 medium (CELLnTEC Advanced Cell System AB; Bern, Switzerland), tailored for both mice and humans, supplemented with serum and growth factors. The lung tissue was clipped along the surface of the lung lobe and cut into 1 mm tissue blocks. Stick the tissue block to the bottom of the culture bottle and add 10 ml of CNT-17 medium to the bottle. The culture bottle was placed vertically in the incubator and then inverted after 4 h, so that the tissue blocks were fully exposed to the medium for culture. The complete epithelial medium was refreshed at four-day intervals.

### Cell culture and transfection

Both AECII and RLE-6TN were cultured with 1640 medium containing 10% fetal bovine serum and 1% Penicillin-Streptomycin. When the cell density reached about 80%∼90% at the bottom of the petri dish, cell digestion and passage could be carried out. First, the old medium was sucked and discarded, washed twice with PBS, and the cells were gently blown away by pipetting PBS, and then the PBS was discarded and appropriate pancreatic enzymes were added to digest the cells. When the cells began to round under the microscope, pancreatic enzymes were discarded immediately and digestion was terminated by adding a medium containing serum. Cells were blown off the bottom of the petri dish with a pipette, then cell suspension was drawn into a centrifuge tube and placed in a centrifuge for centrifugation (rotational speed: 1500 r/min; Time: 5 min). After centrifugation, supernatant was abandoned, and 1640 medium containing 10% fetal bovine serum was added for re-suspension, and the culture dish was planted in the passage ratio of 1:4.

### Protein isolation and Western blotting

Cells and lung tissue were lysed with a precooled lytic mixture of RAPI lysis buffer (1% NP-40, 0.5% Sodium deoxycholate, 0.1% SDS), phosphatase inhibitors (Roche, Basel, Switzerland, 539,142), and inhibitor cocktail (Calbiochem, San Diego, CA, 539,131). After lysis and centrifugation, the supernatant was collected and the protein concentration was measured with BCA assay kit (Thermo, Waltham, MA, 23,225). An appropriate amount of SDS-PAGE loading buffer (Servicebio, Wuhan, China, G2013) was added into the supernatant, which was heated and frozen in the refrigerator for subsequent experiments. Western blotting procedures have been described before [48]. Protein extracts were electrophoresed on 7.5–12.5% SDS polyacrylamide gels at 120 V for 90 min. Subsequently, the separated proteins were transferred onto PVDF membranes (Roche, 49,916,800) using a constant current of 280 mA for 90 min. The PVDF membranes were subjected to overnight incubation at 4 °C with primary antibodies. Following primary antibody exposure, the membranes underwent a subsequent 1-hour incubation with the corresponding HRP-conjugated secondary antibody. Detection of protein signals was achieved through HRP activity-based signal detection methodology. The corresponding primary and secondary antibodies were used to incubate the PVDF membranes, and finally the density of each band was measured by AlphaEaseFC software (Alpha Innotech). The listed antibodies were used in the Western blot as primary antibodies. Anti-BMAL1 (1:1000, ab3350) antibody was abtained from Abcam (Cambridge, UK). Anti-DEC1 (1:1000, 17895-1-AP), anti-fibronectin (1:1000, 15613-1-AP), anti-α-SMA (1:2000, 14395-1-AP), anti-p53 (1:1000, 10442-1-AP), anti-p21 (1:1000, 10355-1-AP), anti-CDK6 (1:1000, 14052-1-AP), anti-CDK2 (1:1000, 10122-1-AP), anti-cyclin E1 (1:1000,11554-1-AP) were purchased from Proteintech Technology (Wuhan, China). Anti-collagen-I (1:500, AM7772) was purchased from ABZOOM (Shanghai, China). An anti-GAPDH (60004-1-Ig, Proteintech Technology, Wuhan, China) was used as a loading control (1:10000).

### RNA extraction and quantitative real-time polymerase chain reaction (qRT-PCR)

Following the manufacturer’s instructions, total RNA of RLE-6TN cells and lung tissues was purified using the RNeasy system (Yeasen, Shanghai, China, 19221ES50) and reverse transcribed with a Hiscript@ Q RT SuperMix (Vazyme, Nanjing, China, R223). Transcript quantification was performed using a Cham Q SYBR qPCR Master Mix (Vazyme, Q311). The mRNA expression levels were normalized to endogenous GAPDH and calculated according to the 2-ΔΔCt method. The following specific primers were used: IL-1α forward 5’- GGA GAG CCG GGT GGT GGT G-3’, IL-1α reverse 5’- GGT GCT GAT CTG GGT TGG ATG G-3’; IL-6 forward 5’- CGC AAG AGA CTT CCA GCC AG-3’, IL-6 reverse 5’- ACT GGT CTG TTG TGG GTG GT-3’; TNF-α forward 5’- ATG GGC TCC CTC TCA TCA GTT CC-3’, TNF-α reverse 5’- GCT CCT CCG CTT GGT GGT TTG-3’; MMP9 forward 5’- GCT CCT CCG CTT GGT GGT TTG-3’, MMP9 reverse 5’- CTG CTT GCC CAG GAA GAC GAA G-3’; BMAL1 forward 5’-GCC CCA CCG ACC TAC TCT-3’, BMAL1 reverse 5’-CTT TGT CTG TGT CCA TAC TTT CTT G-3’; DEC1 forward 5’-GGC TGA TTG AGA AAA AGA GAC G-3’, DEC1 reverse 5’-AGT AAG TTT GAG ATG TTC GGG T-3’; CLOCK forward 5’-CAC TCT CAC AGC CCC ACT GTA-3’, CLOCK reverse 5’-CCC CAC AAG CTA CAG GAG CAG-3’; p21 forward 5’-ATG TCC AAT CCT GGT GAT GTC-3’, p21 reverse 5’-GAA GTC AAA GTT CCA CCG TTC-3’; Cyclin D1 forward 5’-CGT ATC TTA CTT CAA GTG CGT G-3’, Cyclin D1 reverse 5’-ATG GTC TCC TTC ATC TTA GAG G-3’.

### Immunohistochemistry (IHC)

The lung tissues of mice were subjected to fixation with 4% paraformaldehyde and subsequently embedded in paraffin for the purpose of conducting immune-histochemistry. The resulting paraformaldehyde-fixed, paraffin-embedded sections, measuring 5 μm in thickness, were subjected to a series of dewaxing, dehydration, and rehydration steps, followed by staining with Masson, Sirius red, and DEC1 antibody, respectively. The stained lung sections were then examined using light microscopy, which was connected to a digital camera. The extent of collagen deposition was evaluated using Masson trichrome staining, and quantified as a percentage of the surface area stained, using ImageJ software.

### Immunofluorescence staining

Prior to further treatment, cells were seeded on coverslips. Following this, cells were fixed with 4% paraformaldehyde for 15–20 min and subsequently blocked with 5% Bovine serum albumin (BSA, Servicebio, Wuhan, China, G5001-100G) in PBS for 1 h. Primary antibodies against p21 (1:200, Proteintech, 10355-1-AP), p53 (1:200, Proteintech, 60283-2-Ig), and SFTPC (1:200, Proteintech, 10774-1-AP) were then incubated with the cells overnight. The following day, cells were treated with Cy3-labeled secondary antibody (1:200, Servicebio, GB21303) for 1 h. The nuclei in HBEs were stained with DAPI (Servicebio, G1012) for 10 min. Finally, fluorescence images were obtained using a laser-scanning confocal microscopy (LSM 780, Zeiss, Jena, Germany).

### Masson staining

Masson staining was carried out using the Masson Stain Kit (Beyotime, shanghai, China) according to the manufacturer’s instructions. The lung tissue sections were moistened with distilled water for 30–60 s prior to staining. Following wetting, the sections were treated with appropriate volume of hematoxylin nuclear staining solution and fuchsin acid staining solution for 60 s in turn, and washed with distilled water for 30–60 s after each step. Phosphomolybdic acid solution was then applied and left for 6–8 min before being discarded. Aniline blue re-staining solution was then added in an appropriate amount for 5 min, followed by discarding and washing with anhydrous ethanol. After drying, the sections were sealed and observed under a microscope. Quantitative analysis of fibrosis area (%) in tissue sections from each group was performed using ImageJ image analysis software.

### Sirius red staining

The lung sections, measuring 3 μm in thickness, underwent a dewaxing process in xylene, followed by a gradual hydration utilizing a series of ethanol solutions of increasing concentrations. Subsequently, the slides were subjected to staining with Sirius Red for a duration of one hour, after which they were rinsed thoroughly twice with distilled water. Dehydration was achieved through a sequential treatment with 95% and 100% ethanol, succeeded by a clearing step in xylene. Finally, the slides were meticulously sealed with neutral gum to preserve the staining.

### Animal model of pulmonary fibrosis and circadian clock disorder

This study was approved by the Animal Care and Use Committee of Tongji Medical College, Huazhong University of Science and Technology. C57BL/6J mice (Male, 8 weeks) were purchased from Bainite Biotechnology Co (Hubei, China). Bhlhe40 conditional knockout mice were generated using the Cre-loxP system. The Bhlhe40-flox and Sftpc-Cre mice (C57BL/6 N background) were identified and purchased from Cyagen Biosciences (Cyagen, Guangzhou, China). The same loxp sites were inserted on both sides of exon 4 of Bhlhe40 using homologous recombination. When bred with mice harboring cre recombinase, progeny carrying both the Bhlhe40-Flox gene and cre gene may be generated. The expression of cre recombinase leads to the deletion of exons 4 of Bhlhe40 in type 2 alveolar epithelium. The following primers were used: Sftpc-CreERT2: F1: 5’-TGCTTCACAGGGTCGGTAG-3’, R1: 5’-ACACCGGCCTTATTCCAAG-3’; Wildtype: F1: 5’-TGCTTCACAGGGTCGGTAG-3’, R2: 5’-CATTACCTGGGGTAGGACCA-3. KO allele: F1: 5’-CCTGAATAGCCACCAGG TATCTT-3’, R1: 5’-CTGTGATTCTCATTCTAGCCTTGGA-3’; Wild type: F1: 5’-AAGAAA GAAACGCAACACATTGGG-3’, R2: 5’-CTGTGATTCTCATTCTAGCCTTGGA-3’. All mice were kept in specific pathogen-free (SPF) facilities with a 12-hour light/dark cycle, at a temperature of 22 ± 1 °C, and had unrestricted access to food and water.

The mouse pulmonary fibrosis animal model was established as previously described [49]. Bleomycin powder was diluted to 5 mg/ml with 0.9% saline, and then injected into mice by intraperitoneal injection at a dose of 50 mg/kg on days 1, 5, 8, 11, and 15. Mice in the control group were injected with the same amount of saline. After 40 days, all mice were euthanized. The lung tissue was taken for subsequent experiments.

In a model of the mice circadian clock, mice were applied with separate lighting regimens. The adult mice were housed in polyacrylic cages and maintained on a 12 h light:12 h dark cycle for 2 weeks, which made mice synchronized to daily light. Then the chronic jet lag (CJL) group was administered by advancing light onset eight hours in every 48 h. The unshifted control group (LD) was maintained in the LD 12:12. Experimental chronic jet lag or normal circadian rhythm was induced for 8 weeks.

### Cell cycle analysis

The cell cycle was detected using the Cell Cycle and Apoptosis Analysis Kit (Beyotime, C1052) by flow cytometry. MG-63, MNNG/HOS and K7M2 cells were seeded in a 6-well plate at 1 × 105 cells/mL and adhered overnight. The cells were treated with DFO or DFX (0, 12.5, 25, 50, 100 µM) for 24 h. Cells were rinsed with pre-cooled 1× PBS and then trypsinized and collected. The cells were recentrifuged at 1000× g for 3 min, washed once with cold 1× PBS, resuspended in 1 mL of pre-chilled 70% ethanol and stored at 4 °C for 12 h. After washing the cells with cold PBS, 500 µL of PI staining solution was added to each sample and incubated for 30 min at 37 °C in the dark. The samples were tested with a FACSCalibur flow cytometer (BD Biosciences, San Jose, CA, USA), and ModFit software was used to calculate the percentage of cells in each stage of the cell cycle.

### β-Galactosidase staining

The Cell Signaling Senescence β-Galactosidase Staining Kit (Beyotime, Shanghai, China, C0602) was utilized to perform β-galactosidase staining. The experimental procedure involved seeding 2 × 105 cells in each well of 6 well plates and treating them as specified. The cells were cultured until the time of staining. To quantify the number of β-galactosidase staining positive cells, blue positive cells in at least three randomly selected fields at 200× magnification under an inverted microscope were enumerated.

### Chromatin immunoprecipitation (CHIP)

ChIP assays were carried out according to the instructions of the BeyoChIP™ ChIP Assay Kit (Beyotime, Shanghai, China, P2080S). Briefly, the cells were subjected to fixation in 1.0% v/v formaldehyde for 15–20 min at room temperature, followed by quenching with 100 mM glycine. The cells were subsequently disrupted using a mini beadbeater, and the crosslinked chromatin was fragmented by sonication using a Bioruptor (7 cycles of 30 s on/off). The resulting extracts were then incubated for either 2 h or overnight at 4 ˚C with magnetic Prot A beads that were conjugated with a polyclonal antibody against DEC1 (Proteintech, Wuhan, China, 17895-1-AP). Sub-sequently, the reverse cross-linking process was conducted in Tris-EDTA buffer consisting of 100 mM Tris pH 8.0, 10 mM EDTA, and 1.0% v/v SDS at a temperature of 65 °C overnight. Following a proteinase K treatment of 2 h, the samples were subjected to a cleanup process, and the enrichment of DEC1 was determined through real-time PCR using SYBR green mix. The chromatin DNAs that were precipitated underwent Quantitative PCR using the identical procedure as that of qRT-PCR. The percentage of input DNA was utilized to determine the relative enrichment of the DNAs.

### Statistical analysis

In the course of experimentation utilizing RLE-6TN cells and AECII, the experimental and control groups were subjected to identical passage numbers and comparable densities to ensure the consistency of experimental conditions. In animal studies, the allocation of mice to either the experimental or control group was randomized. Statistical analysis of results was conducted using Prism software version 8, employing unpaired t-tests, one-way ANOVA tests, or two-way ANOVA tests. All data are presented as the mean ± SEM, with a *p*-value of less than 0.05 considered significant.

### Electronic supplementary material

Below is the link to the electronic supplementary material.

Supplementary Material 1

## Data Availability

No datasets were generated or analysed during the current study.

## Abbreviations

AT2: Alveolar epithelial type II cell
α-SMA: Alpha-smooth muscle actin
CJL: Chronic jet lag
DEC1: Differentiated embryonic chondrocyte expressed gene 1
ECM: Extracellular matrix
IPF: Idiopathic pulmonary fibrosis
SASP: Senescence-associated secretory phenotypes

## References

[1] Raghu G, Remy-Jardin M, Richeldi L, Thomson CC, Inoue Y, Johkoh T (2022). Idiopathic pulmonary fibrosis (an update) and progressive pulmonary fibrosis in adults: an Official ATS/ERS/JRS/ALAT Clinical Practice Guideline. Am J Respir Crit Care Med.

[2] Parimon T, Yao C, Stripp BR, Noble PW, Chen P. Alveolar epithelial type II cells as drivers of lung fibrosis in idiopathic pulmonary fibrosis. Int J Mol Sci. 2020; 21. 10.3390/ijms21072269 PMC7177323 32218238

[3] Mora AL, Rojas M, Pardo A, Selman M (2017). Emerging therapies for idiopathic pulmonary fibrosis, a progressive age-related disease. Nat Rev Drug Discov.

[4] Reyfman PA, Walter JM, Joshi N, Anekalla KR, McQuattie-Pimentel AC, Chiu S (2019). Single-cell transcriptomic analysis of human lung provides insights into the Pathobiology of Pulmonary Fibrosis. Am J Respir Crit Care Med.

[5] Yao C, Guan X, Carraro G, Parimon T, Liu X, Huang G et al. Senescence of alveolar type 2 cells Drives Progressive Pulmonary Fibrosis. Am J Respir Crit Care Med. 2019. 10.1164/rccm.202004-1274OC PMC7958503 32991815

[6] Roenneberg T, Merrow M (2016). The circadian clock and Human Health. Curr Biol.

[7] Fishbein AB, Knutson KL, Zee PC. Circadian disruption and human health. J Clin Invest. 2021; 131. 10.1172/JCI148286 PMC8483747 34596053

[8] Yu S, Tang Q, Chen G, Lu X, Yin Y, Xie M (2022). Circadian rhythm modulates endochondral bone formation via MTR1/AMPKbeta1/BMAL1 signaling axis. Cell Death Differ.

[9] Sato F, Kohsaka A, Bhawal UK, Muragaki Y. Potential roles of Dec and Bmal1 genes in Interconnecting Circadian Clock and Energy Metabolism. Int J Mol Sci. 2018; 19. 10.3390/ijms19030781 PMC5877642 29518061

[10] Albrecht U (2012). Timing to perfection: the biology of central and peripheral circadian clocks. Neuron.

[11] Mukherji A, Bailey SM, Staels B, Baumert TF (2019). The circadian clock and liver function in health and disease. J Hepatol.

[12] Zhang Z, Zeng P, Gao W, Zhou Q, Feng T, Tian X (2021). Circadian clock: a regulator of the immunity in cancer. Cell Commun Signal.

[13] Chaix A, Lin T, Le HD, Chang MW, Panda S. Time-restricted feeding prevents obesity and metabolic syndrome in mice lacking a circadian clock. Cell Metab. 2019; 29: 303– 19 e4. 10.1016/j.cmet.2018.08.004 PMC7751278 30174302

[14] Patke A, Young MW, Axelrod S (2020). Molecular mechanisms and physiological importance of circadian rhythms. Nat Rev Mol Cell Biol.

[15] Mattis J, Sehgal A (2016). Circadian rhythms, Sleep, and disorders of Aging. Trends Endocrinol Metab.

[16] Zhang W, Xiong Y, Tao R, Panayi AC, Mi B, Liu G (2022). Emerging insight into the role of Circadian Clock Gene BMAL1 in Cellular Senescence. Front Endocrinol (Lausanne).

[17] Li L, Zhang M, Zhao C, Cheng Y, Liu C, Shi M (2022). Circadian clock gene Clock-Bmal1 regulates cellular senescence in chronic obstructive pulmonary disease. BMC Pulm Med.

[18] Yuan G, Hua B, Cai T, Xu L, Li E, Huang Y (2017). Clock mediates liver senescence by controlling ER stress. Aging.

[19] Krishnan N, Rakshit K, Chow ES, Wentzell JS, Kretzschmar D, Giebultowicz JM (2012). Loss of circadian clock accelerates aging in neurodegeneration-prone mutants. Neurobiol Dis.

[20] Sun Q, Zhao Y, Yang Y, Yang X, Li M, Xu X (2017). Loss of the clock protein PER2 shortens the erythrocyte life span in mice. J Biol Chem.

[21] Khapre RV, Kondratova AA, Susova O, Kondratov RV (2011). Circadian clock protein BMAL1 regulates cellular senescence in vivo. Cell Cycle.

[22] Dudek M, Swift J, Meng QJ (2023). The circadian clock and extracellular matrix homeostasis in aging and age-related diseases. Am J Physiol Cell Physiol.

[23] Heo JW, Lee HE, Lee J, Choi LS, Shin J, Mun JY et al. Vutiglabridin alleviates Cellular Senescence with metabolic regulation and circadian clock in human dermal fibroblasts. Antioxid (Basel). 2024; 13. 10.3390/antiox13010109 PMC10812742 38247533

[24] Ding SL, Zhang TW, Zhang QC, Ding W, Li ZF, Han GJ (2021). Excessive mechanical strain accelerates intervertebral disc degeneration by disrupting intrinsic circadian rhythm. Exp Mol Med.

[25] Sato F, Bhawal UK, Yoshimura T, Muragaki Y (2016). DEC1 and DEC2 crosstalk between Circadian Rhythm and Tumor Progression. J Cancer.

[26] Bhawal UK, Sato F, Arakawa Y, Fujimoto K, Kawamoto T, Tanimoto K (2011). Basic helix-loop-helix transcription factor DEC1 negatively regulates cyclin D1. J Pathol.

[27] Kato Y, Kawamoto T, Fujimoto K, Noshiro M (2014). DEC1/STRA13/SHARP2 and DEC2/SHARP1 coordinate physiological processes, including circadian rhythms in response to Environmental Stimuli. Curr Top Dev Biol.

[28] Wang Y, Guo H, He F (2023). Circadian disruption: from mouse models to molecular mechanisms and cancer therapeutic targets. Cancer Metastasis Rev.

[29] Sundar IK, Yao H, Sellix MT, Rahman I (2015). Circadian molecular clock in lung pathophysiology. Am J Physiol Lung Cell Mol Physiol.

[30] Sundar IK, Sellix MT, Rahman I (2018). Redox regulation of circadian molecular clock in chronic airway diseases. Free Radic Biol Med.

[31] Hadden H, Soldin SJ, Massaro D (2012). Circadian disruption alters mouse lung clock gene expression and lung mechanics. J Appl Physiol (1985).

[32] Lu Y, Liu B, Ma J, Yang S, Huang J (2021). Disruption of Circadian Transcriptome in Lung by Acute Sleep Deprivation. Front Genet.

[33] Taylor L, Von Lendenfeld F, Ashton A, Sanghani H, Di Pretoro S, Usselmann L (2023). Sleep and circadian rhythm disruption alters the lung transcriptome to predispose to viral infection. iScience.

[34] Cunningham PS, Meijer P, Nazgiewicz A, Anderson SG, Borthwick LA, Bagnall J (2020). The circadian clock protein REVERBalpha inhibits pulmonary fibrosis development. Proc Natl Acad Sci U S A.

[35] Confalonieri P, Volpe MC, Jacob J, Maiocchi S, Salton F, Ruaro B et al. Regeneration or repair? The role of alveolar epithelial cells in the pathogenesis of idiopathic pulmonary fibrosis (IPF). Cells. 2022; 11. 10.3390/cells11132095 PMC9266271 35805179

[36] Schafer MJ, White TA, Iijima K, Haak AJ, Ligresti G, Atkinson EJ (2017). Cellular senescence mediates fibrotic pulmonary disease. Nat Commun.

[37] Hansel C, Jendrossek V, Klein D. Cellular Senescence in the lung: the Central Role of senescent epithelial cells. Int J Mol Sci. 2020; 21. 10.3390/ijms21093279 PMC7247355 32384619

[38] Huang W, Hickson LJ, Eirin A, Kirkland JL, Lerman LO (2022). Cellular senescence: the good, the bad and the unknown. Nat Rev Nephrol.

[39] Qu M, Zhang G, Qu H, Vu A, Wu R, Tsukamoto H (2023). Circadian regulator BMAL1::CLOCK promotes cell proliferation in hepatocellular carcinoma by controlling apoptosis and cell cycle. Proc Natl Acad Sci U S A.

[40] Yao J, He C, Zhao W, Hu N, Long D (2021). Circadian clock and cell cycle: Cancer and chronotherapy. Acta Histochem.

[41] Laranjeiro R, Tamai TK, Letton W, Hamilton N, Whitmore D (2018). Circadian clock synchronization of the cell cycle in zebrafish occurs through a gating mechanism rather than a period-phase locking process. J Biol Rhythms.

[42] Shostak A, Ruppert B, Ha N, Bruns P, Toprak UH, Project IM-S (2016). MYC/MIZ1-dependent gene repression inversely coordinates the circadian clock with cell cycle and proliferation. Nat Commun.

[43] Rauschmeier R, Gustafsson C, Reinhardt A, A-Gonzalez N, Tortola L, Cansever D et al. Bhlhe40 and Bhlhe41 transcription factors regulate alveolar macrophage self‐renewal and identity. EMBO J. 2019; 38. 10.15252/embj.2018101233 PMC6769426 31414712

[44] Zafar A, Ng HP, Kim GD, Chan ER, Mahabeleshwar GH (2021). BHLHE40 promotes macrophage pro-inflammatory gene expression and functions. FASEB J.

[45] Park SL, Mackay LK (2019). Bhlhe40 keeps Resident T cells too fit to quit. Immunity.

[46] Li CF, Zhu BB, Son YM, Wang Z, Jiang L, Xiang M (2019). The transcription factor Bhlhe40 Programs Mitochondria! Regulation of Resident CD8(+) T cell fitness and functionality. Immunity.

[47] Khan P, Fytianos K, Tamo L, Roth M, Tamm M, Geiser T (2018). Culture of human alveolar epithelial type II cells by sprouting. Respir Res.

[48] Chen SJ, Huang Y, Yu F, Feng X, Zheng YY, Li Q (2023). BMAL1/p53 mediating bronchial epithelial cell autophagy contributes to PM2.5-aggravated asthma. Cell Commun Signal.

[49] Zhang Q, Ye H, Xiang F, Song LJ, Zhou LL, Cai PC (2017). miR-18a-5p inhibits sub-pleural pulmonary fibrosis by targeting TGF-beta receptor II. Mol Ther.

