# Supplementary material for: DEC1 is involved in circadian rhythm disruption-exacerbated pulmonary fibrosis
Source: Cell Commun Signal. 2024 Apr 26;22:245. doi: 10.1186/s12964-024-01614-w (PMC11046974; doi:10.1186/s12964-024-01614-w)
Supplement: Supplementary file 1 — Supplementary Material 1 [file 12964_2024_1614_MOESM1_ESM.pdf]

Online supplementary materials

Title: DEC1 is involved in circadian rhythm disruption-exacerbated pulmonary fibrosis

Authors: Shuai-Jun Chen et al

Contents

Fig. S1. Electron microscopy image of alveolar epithelial type II cell (AT2)

Fig. S2. Cell senescence protein P21 and P53 levels increased in mouse lung tissues of bleomycin-induced pulmonary fibrosis model

Fig. S3. Knockdown of DEC1 with DEC1 siRNA depressed AT2 cell senescence in bleomycin-induced pulmonary fibrosis model *in vivo*.

Fig. S4. The depletion of p21 exhibited no discernible impact on the expression levels of DEC1 protein.

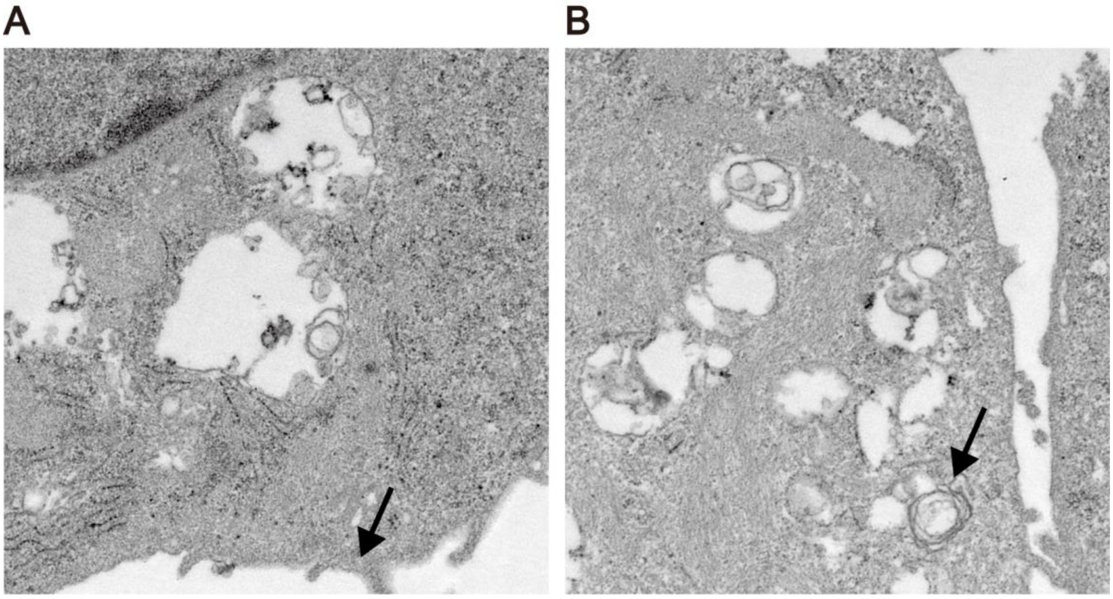

Fig. S1. Electron microscopy image of AT2 cells. (A) microvilli (arrow). (B) lamellar bodies (arrow).

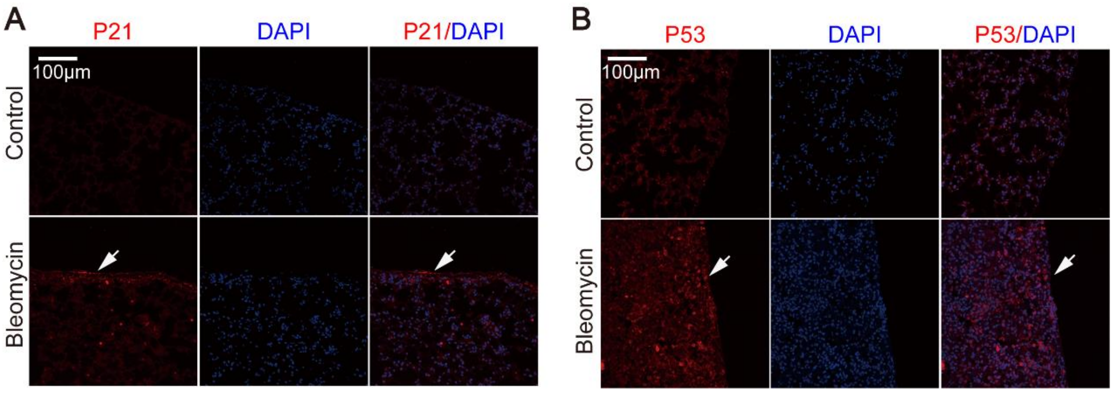

Fig. S2. Cell senescence protein P21 and P53 levels increased in mouse lung tissues of bleomycin-induced pulmonary fibrosis model. (A, B) In mouse pulmonary fibrosis model, the expression levels of p21 and p53 protein in lung tissues were detected by immunofluorescence assay. Original magnification, ×400.

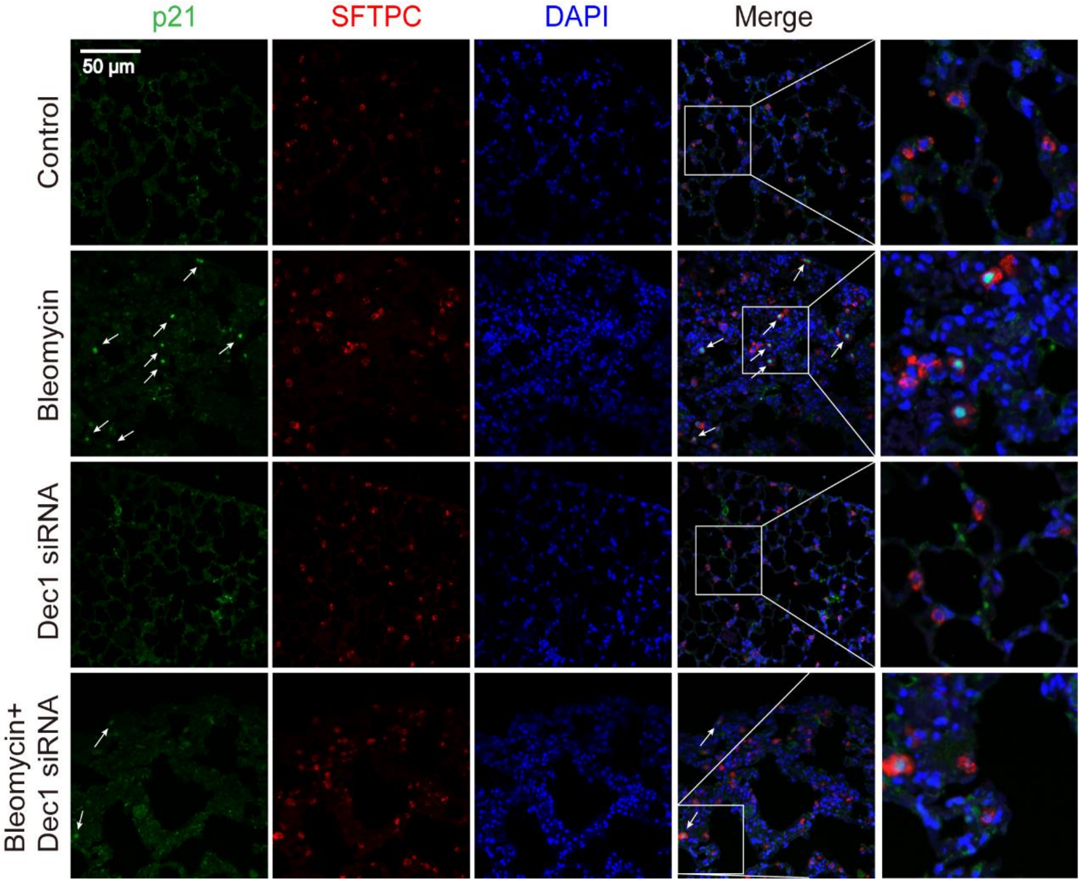

Fig. S3. Knockdown of DEC1 with DEC1 siRNA depressed AT2 cell senescence in bleomycin-induced pulmonary fibrosis model *in vivo*. Representative images of p21 protein expression in lung tissues were detected by immunofluorescence assay in a pulmonary fibrosis model with DEC1 siRNA lentivirus injection (Green: p21, red: SFTPC).

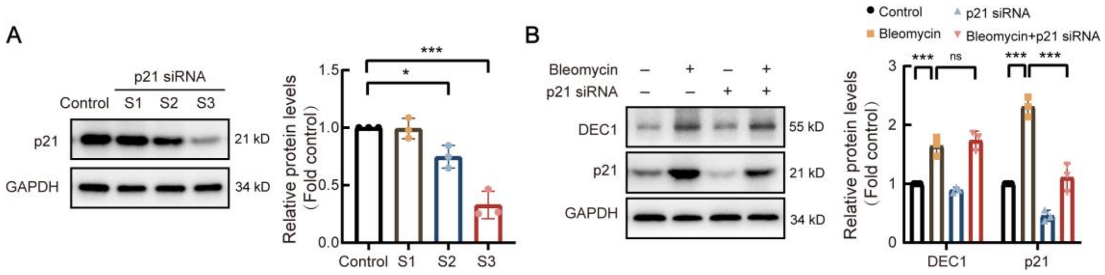

Fig. S4. The depletion of p21 exhibited no discernible impact on the expression levels of DEC1 protein. (A) Three siRNAs targeting p21 were employed to treat alveolar epithelial cells, facilitating the assessment of siRNA-mediated interference efficiency (n=3). (B) RLE-6TN cells were treated with bleomycin and p21 siRNA, and the expression levels of senescence-associated proteins DEC1 and p21 were detected by Western blot analysis (n=3). **P* < 0.05, ****P* < 0.001, ns means nonsignificant.
